# Frequency of specific *agr* groups and antibiotic resistance in *Staphylococcus aureus *isolated from bovine mastitis in the northeast of Iran

**Published:** 2015-12-15

**Authors:** Mohammad Mohsenzadeh, Kiarash Ghazvini, Amir Azimian

**Affiliations:** 1 *Department of Food Hygiene and Aquaculture, Faculty of Veterinary Medicine, Ferdowsi University of Mashhad, Mashhad, Iran; *; 2 *Antimicrobial Resistance Research Center, Avicenna Research Institute, Tehran, Iran;*; 3 *Department of Microbiology and Virology, Faculty of Medicine, Mashhad University of Medical Sciences, Mashhad, Iran;*; 4 *Department of Pathobiology, School of Medicine, North Khorasan University of Medical Sciences, Boujnord, Iran.*

**Keywords:** Antibiotic resistance, Bovine mastitis, Specific *agr* groups, *Staphylococcus aureus*

## Abstract

*Staphylococcus aureus* is generally regarded as a leading cause of mastitis in dairy cattle. The aim of this study was to investigate the pattern of *agr* groups and any possible relationship between *agr* groups and antibiotic resistance among *S. aureus* strains isolated from bovine mastitis in Northeast of Iran. For this purpose, a total of 300 bovine mastitic milk samples were taken from dairy industry farms of Khorasan Razavi Province, Iran. *S. aureus* were isolated and identified according to the standard methods. Antibiotic susceptibility testing was conducted by disk diffusion method. In this study a total of 31 isolates of *S. aureus* were evaluated for *agrD* gene polymorphism by specific primers. Most of the isolates belonged to *agr* group I (54.8%), followed by *agr* group III (25.8%) and *agr* group II (19.4%). There was not any isolates belonging to group IV. Resistance to methicillin in *agr* group I isolates was more than other groups. *Agr* groups II and III were quite susceptible to methicillin. Due to high prevalent of *S. aureus* isolates and high antibiotic resistance rate in bovine mastitic isolates, it is important to verify the characteristics of *S. aureus* strains in Iran.

## Introduction

*Staphylococcus aureus* is a common cause of subclinical mastitis worldwide, which is of economic importance to the dairy industry.^1^ The main reservoir of *S. aureus* in milk and milk products seems to be infected quarter. Molecular epidemiological analysis of the bovine *S. aureus* population suggested that a small number of clonal types were responsible for most infections, and that isolates had a broad geographic distribution.^2^^-^^4^ *Staphylococcus aureus* has a capacity to produce a large number of putative virulence factors. Some of these factors may be more important than others in different diseases or at different stages of the pathogenesis of particular infections, as not all factors are produced by each strain.^5^^-^^7^

Recently the accessory gene regulator (*agr*) locus was identified as a regulator of virulence factors in* S. aureus*. It controls a large set of genes including those encoding cell wall associated and extracellular proteins.^8^

The *agr* locus is composed of two divergent transcriptional units, RNAII and RNAIII driven by the P2 and P3 promoters respectively. The P2 operon encodes four proteins (*agrA, agrB, agrC *and *agrD*) that generate the *agr*-sensing mechanisms. The *agr* signaling system consists of a classical two-component regulatory system in which the *agrC* (the signal receptor) bind the extracellular auto inducing peptide, *agrD* (AIP) and modulates the activity of *agrA*, the response regulator*. agrA* activity then leads to greatly increased P2 and P3 transcription. *agrB* is a transmembrane protein that involved in processing and secreting of the *agrD*. Sequence diversity in the variable region, comprised of the last one-third of *agrB, agrD* and the first half of *agrC*, has generated the four *agr* specificity group in *S. aureus*. The AIP produced by a given strain of *S. aureus* activates its own *agr* locus but may inhibit the expression of *agr *in other isolates. This AIP-dependent inhibition is correlated with ability of a strain to compete with other isolates for sites of infection. RNAIII encodes several virulence factors, including TSS toxin1 and hemolysins. The Staphylococcal *agr *system also decreases the expression of several cell wall-associated proteins.^8^^-^^10^

 In this research we study the pattern of *agr* groups among *S. aureus* isolates obtained from bovine mastitis and investigate any possible relationship between *agr* groups and antibiotic resistance in this region.

## Materials and Methods

**Bacterial isolates. **Milk samples (10 mL) were taken aseptically from all quarters of 300 bovine infected udders of some dairy industry farms of Khorasan Razavi, Northeast of Iran. The presence of *Staphylococcus *spp. was determined by culturing 0.01 mL of each sample on 5% bovine blood agar (Merck Millipore, Darmstadt, Germany) plates and incubated at 37 ˚C for 24 to 48 hr. A quarter was identified as infected when a single pathogenic bacterium was isolated and somatic cell count (SCC) was increased above 200,000 mL^-1^.

The bacterial isolates were presumptively identified on the basis of morphology, catalase production, hemolysis pattern and Gram staining of the colonies, coagulase activity on rabbit plasma (Bio-Merieux, Marcy l'Etoile, France), mannitol fermentation on mannitol salt agar (MSA; Himedia Labs, Mumbai, India), production of clamping factor (Slidex Staph Plus; Bio-Merieux, Marcy l'Etoile, France) and each bacterial isolates was streaked on blood agar to obtain a pure culture. 

**Antimicrobial susceptibility. **Antibiotic susceptibility testing was conducted by disk diffusion method according to the guidelines of the National Committee for Clinical Laboratory Standards.^11^

**DNA extraction and amplification. **The genomic DNA of *S. aureus* isolates was extracted by the modified phenol-chloroform method. Lysates of colonies were prepared according to protocol given by Sharma *et al.* ^12^

The specific *agr* groups were determined by poly-merase chain reaction (PCR) with use of pan forward* agr*: 5'-ATGCACATGGTGCACATGC-3' corresponding to conserved region from *agrB* gene, Reverse *agr *I: 5'-GTCACAAGTACT ATAAGCTGCGAT-3' (in the *agrD *gene, product size 440 bp), Reverse *agr *II: 5'-GTATTACTAATTGAAAAGTGCCATA GC-3' (in the *agrC* gene, product size 572 bp), Reverse *agr *III: 5'-CTGTTGAAAAAGTCAACTAAAAGC TC-3' (in the *agrD* gene, product size 406 bp) and Reverse *agrIV*: 5'-CGATAA TGCCGTAATACCCG-3' (in the *agrC* gene, product size 588 bp).^13^ The PCR assay was performed by adding 1 µL of a 1:200 dilution of *S. aureus* DNA template and 24 µL of water to 25 µL of a PCR mixture that includes 2.5 IU of *Taq* DNA polymerase, 2 mM MgCl_2_, 350 µm dNTPs and 25 mM KCl in 0.2 mL PCR tubes. Thermal cycling was performed in a thermal cycler (Techne, Cambridge, UK) and consisted of 30 cycles of denaturation (94 ˚C in 60 sec), annealing (57 ˚C in 60 sec) and extension (72 ˚C 60 sec). Aliquots of amplified samples were analyzed by electrophoresis on a 1.0% agarose gel and stained with ethidium bromide. *S. aureus* strains RN6390 (*agr* group I), RN6607 (*agr* group II), RN8465 (*agr* group III), RN4550 (*agr* group IV) and RN6911 (*agr* negative) were used as positive control for *agr *group identification, kindly provided by Dr. Richard P. Novick (Skirball Institute of Biomolecular Medicine, New York, USA).

**Statistical analysis. **Statistical differences between groups were analyzed by means of student’s *t*-test or analysis of variance (ANOVA) tests in SPSS (version 17; SPSS Inc., Chicago, USA). Multivariate analysis was performed to assess the independence of the statistically significant variables in unvaraiate analysis. A *p*-value less than 0.05 was considered significant.

## Results

**Fig. 1:**
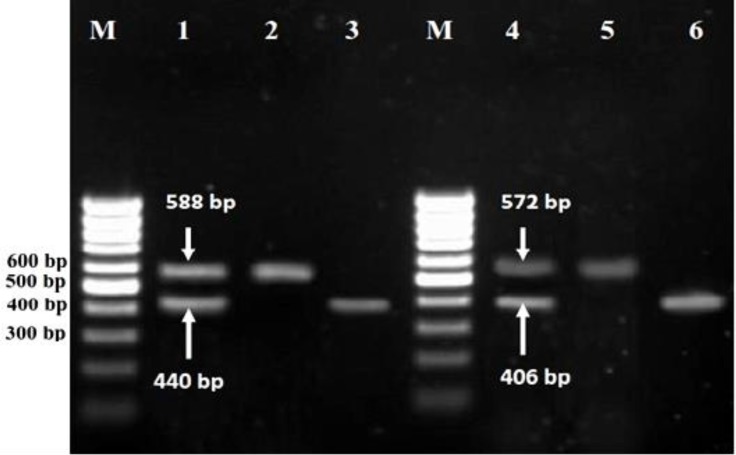
Agarose gel electrophoresis of PCR products for *agr* specific groups. Lane M, 100 bp DNA molecular size marker; Lane 1, Control positive (combined PCR products of RN6390 (440 bp for* agrI *group*,*) and RN4550 (588 bp for* agrIV,*group) reference strains); Lane 2, PCR product of *agrII* group; Lane 3, PCR product of *agrI *group; Lane 4, Control positive (combined PCR products of RN6607 (572 bp for* agrII *group*,*) and RN8465 (406 bp for* agrIII,*group) reference strains); Lane 5, PCR product of *agrII* group and lane 6, PCR product of *agrIII *group

In this study a total of 31 isolates from 300 samples were evaluated. Analysis of *agrD* gene polymorphism by specific primers allowed assigning our isolates in one of four major specific *agr* groups. The *agr* specific groups were determined by mentioned PCR method (Fig. 1).

Most of our isolates belonged to *agr* group I (54.8%), followed by *agr* group III (25.8%) and *agr* group II (19.4%). There was not any strain belonging to group IV. 

Considering the possible relation between the *agr* group and the antibiotic resistance in *S. aureus* isolates, we used disk diffusion agar test according to National Committee for Clinical Laboratory Standards (NCCLS) and the results were demonstrated in Table 1.

According to these results, there is no significant association between resistance against penicillin and *agr* group but there is a significant association between *agr* group and resistance against methicillin.

**Table 1:** Frequency of *agr* groups and their antibiotic resistance among *Staphylococcus aureus* isolates. The values indicate the number (frequency) of isolates.

**Antibiotic**	***agr*** ** groups**			**Total** **(n = 31)**
	***agr*** ** group I** **(n = 17)**	***agr*** ** group II** **(n = 6)**	***agr*** ** group III** **(n = 8)**	
**Penicillin**	15(88.2%)	5(83.3%)	8(100%)	28(90.3%)
**Ampicillin**	12(70.6%)	3(50.0%)	6(75.0%)	21(67.7%)
**Cefalotin**	1(5.9%)	1(16.7%)	1(12.5%)	3(9.7%)
**Cefixime**	8(47.0%)	2(33.3%)	2(25.0%)	12(38.7%)
**Tetracycline**	5(29.4%)	0	1(12.5%)	6(19.4%)
**Gentamycin**	0	0	0	0
**Methicillin**	9(52.9%)	0	0	9(29.0%)
**Vancomycin**	0	0	0	0

## Discussion

*Staphylococcus aureus* is a major pathogen in both human and animals.^14^ The ability of this organism to cause multitude of human disease such as endocarditis, pneumonia, bacteremia and toxic shock syndrome (TSS) and diseases of farm animals such as mastitis suggested that the pathogenesis of *Staphylococcus aureus* infections is highly complex. The virulence of organism is dependent on many cell surface proteins and secreted exotoxins and enzymes and it is suggested that the environmental and host signals are contributing the regulation of virulence factors.^15^ The *agr* operon is involved in the coordinate regulation of some of *Staphylococcus aureus* virulence factors. *Staphylococcus aureus* isolates exhibit well-defined genetic polymorphisms within the *agr* locus. Four *agr* genotypes, group I to IV have been described to date.^16^

Takeuchi *et al.* have shown that among 76 *S. aureus* isolated from cow mastitis in Japan, 43.4% isolates belonged to *agr* group I , 18.4% belonged to *agr* group II and 38.2% belonged to *agr* group III.^17^ This report is in consistent with our findings which showed *agr* group I (54.8%), *agr* group II (19.4%) and *agr* group III (25.8%).

Gilot *et al.* reported that among 71 *S. aureus* isolated from bovine mastitis 69.0% of isolates belonged to *agr* group I , 23.9% belonged to *agr* group II, 2.8% were *agr* group III and 1.4% were belonged to *agr* group IV that were somewhat different from our results and more similar to the human isolates.^18^

Although data relating *agr* type and specific infections are scarce, Jarraud *et al*. have shown that specific *agr* genotype isolates might be associated with particular infectious syndromes.^15^ For example, disease mediated by enterotoxin is linked to *agr* group I, infective endocarditis is linked to *agr* groups I and II, toxic shock syndrome is linked to *agr* group III and exofoliative disease is linked to *agr* group IV. Recently, *agr* group III genotype isolates have been over represented in isolates from community-acquired methicillin-resistant *S. aureus* (MRSA) infections, whereas previous nosocomially isolated MRSA isolates in the United States were predominantly of *agr* group II.^19^^,^^20^ Most exofoliatin producing isolates responsible for staphylococcal scalded skin syndrome (SSSS) belonged to *agr* group IV.^21^

In our study resistance to methicillin in *agr* group I isolates was more than other groups. *Agr* groups II and III were quite susceptible to methicillin. Furthermore, other studies showed a correlation between induction of the glycopeptide intermediate *S. aureus* (GISA) phenotype and autolytic deficiency, especially in the context of the *agr* genotype II,^22^ but in our study all isolates were susceptible to vancomycin. Some reports state that there are clinical trends according to each *agr* group. For example, *agr* group I was prevalent in a collection of 192 *S. aureus* isolates, 71.0% of which were methicillin resistant.^16^^,^^21^

Moise-broder et al. showed that *agr* group II poly-morphism in MRSA predicts the failure of vancomycin therapy.^23^ Reportedly, community-acquired MRSA belonged to *agr* group III and methicillin sensitive *S*. *aureus* (MSSA)^24^ and toxic shock syndrome toxin (TSST-1) producing isolates belonged to the *agr* specificity group III.^17^ Recent data demonstrate that the vast majority of MRSA in France and around the world belong to *agr* group III.^24^^,^^25^

In our study most of MRSA isolates were belonged to *agr* group I and all of MSSA isolates belonged to *agr* groups II and III. *Agr* specific group IV was absent in many previously reported.^13^^,^^16^^,^^26^ and also we do not have any group IV isolates in our isolates. This is more likely due to ecological and geographical structuring. 

There seems to be a geographic difference between *agr* groups. Most isolates belonged to *agr* group I, represented by the Brazilian, Portuguese, Hungarian and Berlin. Group II isolates, represented by the pediatric and Japan isolates, have been isolated mainly in Japan and North America. Isolates of group III isolated mainly in Europe.^27^ Our isolates revealed that group I isolates are prevalent in Iran, followed by group III and group II, which was relatively small compared to the previous groups. Iran is one of several countries with high antibiotic resistance rate. Therefore it is important to verify the characteristics of *S. aureus* in this country. The results of this study showed the status of *agr* groups and antibiotic resistance patterns between *S. aureus* isolated from bovine mastitis in Northeast of Iran. This study may also aid finding an appropriate method to eradicate resistant clones because *agr* is a potential target for therapy and the response can be modulated by the synthetic peptides. 
